# A review of the impact of shelter design on the health of displaced populations

**DOI:** 10.1186/s41018-022-00123-0

**Published:** 2022-08-30

**Authors:** Anna Conzatti, Tristan Kershaw, Alexander Copping, David Coley

**Affiliations:** Department of Architecture and Civil Engineering, University of Bath, Claverton Down, Bat, UK

**Keywords:** Shelter, Health, Bibliometric Analysis, Air quality, Thermal comfort

## Abstract

There are currently millions of displaced people encamped in low-quality shelters that jeopardise the health of these displaced populations. These shelters, which exhibit poor thermal regulation and air quality, are often inhabited by households for several years. Recently, the internal environment of shelters has been recognised as a determinant of the health of the occupants and the indoor air quality (IAQ) and internal temperatures have been identified as critical factors affecting occupants’ health. Attempts by researchers and private companies to develop healthier shelter solutions have mainly prioritised factors such as rapid deployment, transportability and sustainability. Via a systematic bibliometric analysis of the existing literature, this review examines the impact of shelters’ internal environment on occupant health. Self-reports and building simulation are the most common methodologies reported in the literature, but there is a disconnect between the reported shelter issues and their impact on health. This is likely due to the multifaceted and site-specific factors analysed. Indoor air quality, thermal comfort and overcrowding are the most commonly identified shelter issues, which are strongly related to the presence of infectious and airborne diseases. An analysis of the available literature indicates that there is still a lack of clear guidance linking shelter quality to health. Moreover, evidence of the impact of shelters on health is harder to find, and there is a gap regarding the metrics and the methodology used to evaluate shelter quality. Therefore, further research is necessary to provide evidence of the impact of shelter design on health through transdisciplinary approaches.

## Introduction

According to the latest UNHCR report (UNHCR 2019) around 80 million people currently are forcibly displaced as a consequence of conflict or persecution and another 175 million are displaced due to natural disasters. These numbers are the highest since the start of the UNHCR mandate (1951). More than 6.6 million of displaced persons are encamped in areas subjected to extreme climate and their living conditions are exacerbated by the lack of adequate WaSH (Water and Sanitation Hygiene) facilities and shelter quality (UNHCR 2021).

Prolonged exposure to extreme temperatures, poor living conditions, low air quality, the lack of clean water and the risks associated with overcrowding and mass movement expose displaced populations to a higher prevalence of certain diseases. The World Health Organization (WHO) has declared that the poor conditions inside shelters and lack of adequate WaSH facilities are significant contributors to the development and spread of acute respiratory diseases, diarrheal diseases, malaria and measles (World Health Organization 2018a). It has been observed that the most frequent health problems exhibited include: airborne diseases, hypothermia, gastrointestinal disorders, cardiovascular issues and injuries (Dubray and Guha-Sapir 2018). Additionally, the last UNHCR report (WHO 2019) stated that communicable diseases represent 92.3% of all medical consultations and that upper respiratory tract infection (URTI) is the leading cause of morbidity in refugee camps. This further indicates that low indoor air quality (IAQ) within shelters is a significant cause of illness for displaced populations.

The humanitarian sector and host governments play a crucial role in helping to reduce the spread of both physical and mental diseases by facilitating access to proper health care and by providing shelters that have a minimal impact on health. Due to the importance of the issue, it has been a longstanding requirement of humanitarian agencies to provide healthy and safe shelters for the population.

The influence of housing on human health has long been recognised (Bonnefoy et al. 2003; Shaw 2004; Bonnefoy 2007), and there is a considerable body of evidence to demonstrate the correlation between the indoor environmental quality (IEQ) and the health of households. However, the definition of 'healthy housing' remained blurred until 2018 when the WHO released its first set of guidelines linking housing and health (World Health Organization 2018b). These guidelines followed an increasing number of studies developed over the last two decades which focused on ‘healthy housing’. For example, Shaw (2004) demonstrated that housing is a determinant for health and poor housing quality has a major impact on respiratory diseases, due to temperature, humidity and ventilation. Matte and Jacobs (Matte and Jacobs 2000) suggested a range of factors that characterise healthy housing such as low levels of indoor air pollutants, protection against extreme temperatures, or absence of moisture and fungi (mould) growth. The literature also consolidates the link between housing and mental health (Owoaje et al. 2016; Cardozo and Mollica 2018; Ziersch and Due 2018; Wu et al. 2019). In these studies, evidence was presented showing a relationship between overcrowding and mental health (including stress and sleep disorders) and between indoor temperatures and mental health disorders.

A growing body of research focuses on the intersections between housing and health, but there is still a lack of research concerning the impact of shelter quality on displaced populations (Ziersch and Due 2018; Shackelford et al. 2020; Behnke et al. 2020). Furthermore, there is a lack of evidence supporting the link between shelter performances and the health of displaced populations. This is due to the hugely challenging and unpredictable conditions that do not facilitate accessibility to collect quality health data or the possibility to monitor the situation for a prolonged period (World Health Organization 2018a).

In order to compare studies on shelter quality and health, a preliminary understanding of the vocabulary used to define shelter as a noun and not as a process is needed. The humanitarian sector uses a great deal of terminology in the description of ‘shelter’ and often, these terms can make it difficult to define what the noun ‘shelter’ means (Alshawawreh et al. 2020). Generally, the language used to define a shelter may be confusing as the terms ‘shelter’, ‘housing’ and ‘temporary housing’ are often used interchangeably. Typically, the term ‘shelter’ as a noun describes a basic emergency and temporary condition. While 'housing' refers to a recovery process on a longer-term. Therefore, the term ‘shelter’ is often inappropriately used in association with recovery. Different terms are used to indicate similar types of accommodation and definitions and roles often overlap. What remains common though is the right to a suitable structure for human habitation, which can ensure displaced families can live with dignity and safety.

In the existing literature, the aforementioned terms have been often used interchangeably to describe the same type of accommodation, nevertheless, this review will use only the term shelter, to avoid ambiguity. Furthermore, for the purposes of this paper, the term shelter design has been broadly defined, encompassing the shelter characteristics having a direct impact on shelter’s indoor environment, such as the physical structure of the shelter, the layout, the materials used for the construction, the ventilation strategy, the heating strategy and overcrowding, but not WaSH facilities. Studies focused on the impact of lack of presence of WaSH facilities have been excluded.

In order to conduct a comprehensive review of the literature covering shelters and health, a systematic bibliometric study of the literature, followed by a comparative analysis was undertaken to identify:

1. What are the shelter design guidelines related to health?
2. What are the health outcomes of poor shelter design?
3. What are the main factors affecting refugee’s health concerning shelter design?
4. How have these factors been investigated in previous studies?

## Systematic and bibliometric review

A systematic review with the support of bibliometric network analysis was initially conducted to describe how the impact of shelter on displaced people’s health is assessed and evaluated within the design process and the provision of shelters. The review specifically examines refugee populations and internally displaced people (IDPs), but not homeless populations or migrants. The selected articles focus on the relationship between shelter quality and human health. Peer-reviewed studies were identified from three databases: Scopus, Web of Science and PubMed. Additional grey literature have been idenfied from shelter-relevant portals: the United Nation High Commissioner for Refugees (UNHCR), the International Committee of the Red Cross (IFRC) and Shelter Cluster.

The research strategy was based on a preliminary reading of the existing literature focusing on housing and health in displacement. The UNHCR Handbook of Emergencies (UNHCR 2007) and the Sphere Handbook (Sphere Association 2018), the two main manuals for displaced settings, have been used as source of research terms. However, the definition of the initial list of keywords presented some limitations since an agreed terminology defining what shelter is, does not exist (Alshawawreh et al. 2020).

Quarantelli (Quarantelli 1982), for instance, adopted a shelter terminology based on four different housing phases in post-disaster reconstruction, resulting in emergency shelter, temporary shelter, temporary housing and permanent housing. The UNHCR and IFRC, the two primary shelter providers, also developed their terminology to categorise shelters. UNHCR (UNHCR 2016) defined four shelter categories according to the lifespan and the materials used: global shelter, emergency shelter, transitional shelter and durable shelter. According to the length of stay, permanency of the location, expected lifespan and the material used, IFRC (IFRC 2013) identified seven categories of shelter: emergency shelters, temporary shelters, temporary housing, transitional shelters and core shelters. As can be observed, shelter and house/housing are the most frequent terms mentioned in the literature. Thus, they have been selected as search terms. The terms ‘health’, ‘refugee’ and ‘IDP’ have been defined more broadly for the purpose of the research.

The analysis included articles published and available online up to the beginning of 2021.

**Fig. 1:**
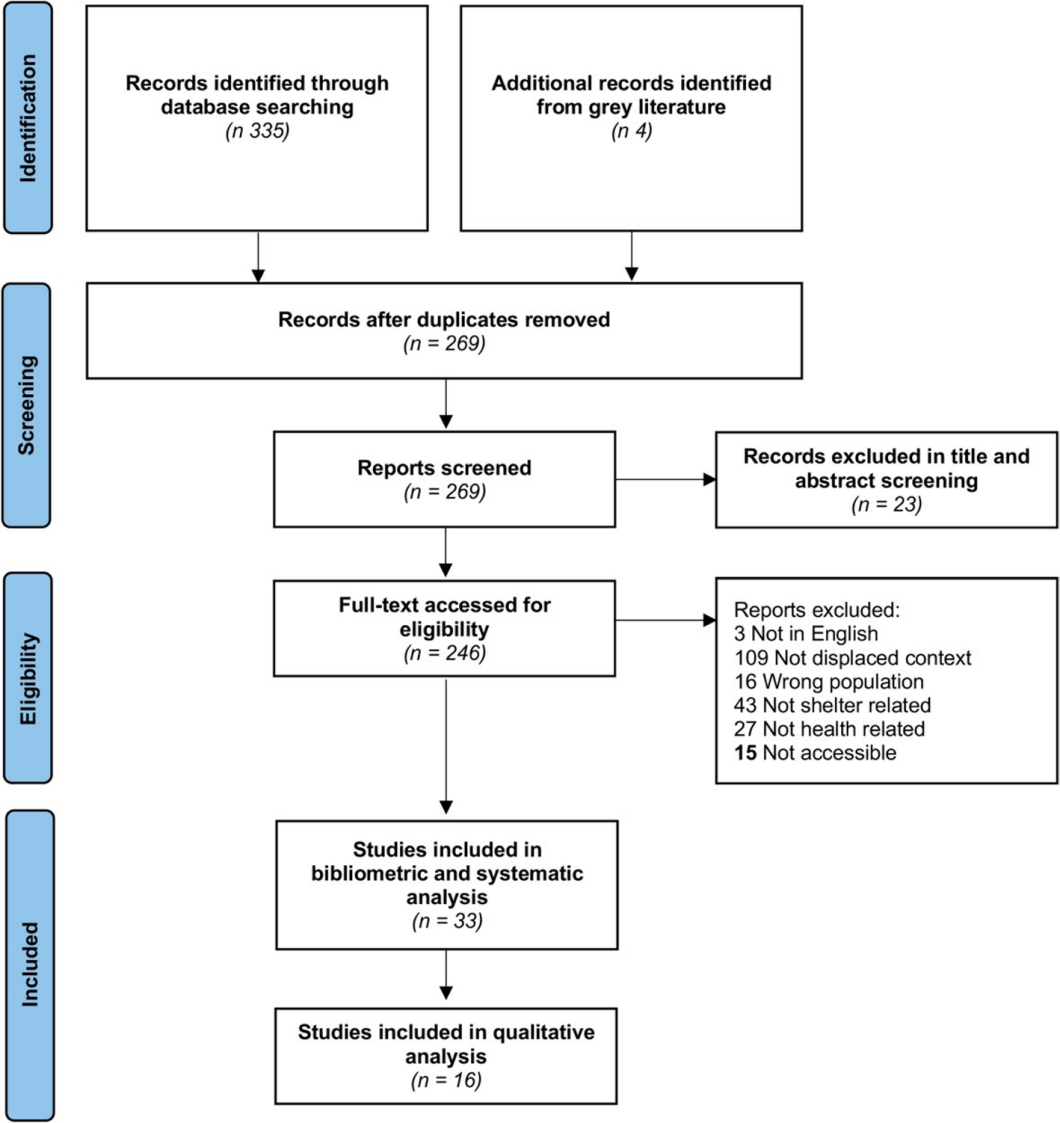
Flow diagram for a systematic review on shelter and health

The initial search, as shown in Fig. 1, returned 269 results that were considered for inclusion. All of the selected papers were downloaded, and the abstracts were reviewed to meet inclusion criteria. Eligibility criteria included papers written in English and studies focusing on refugees, and/or internally displaced and displaced populations. Studies focusing on migrants only were excluded because the term ‘migrant’ often does not give sufficient clarity on the background of participants (Ziersch et al. 2017; Ziersch and Due 2018). Out of 269 papers, 33 publications that met the inclusion criteria were identified.

### Bibliometric analysis

**Fig. 2:**
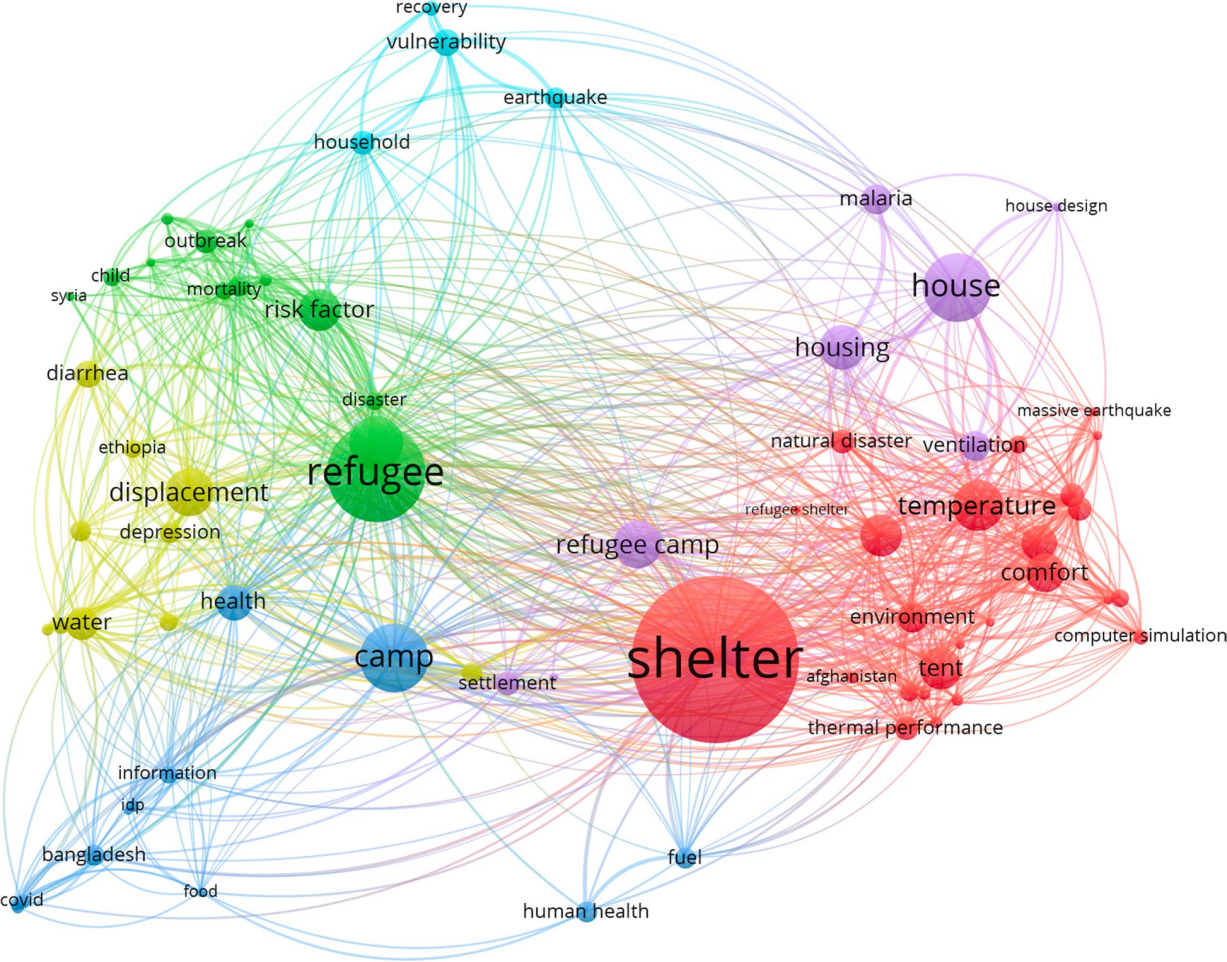
VOS viewer co-occurrence network visualisation mapping of the most frequent keywords (minimum of three occurrences) in shelter and health

Full texts were obtained, reviewed and subjected to bibliometric analysis. Bibliometric analysis is a statistical method which can analyse the critical areas of research and predict the direction of future studies. Vos Viewer (van Eck and Waltman 2010) allows the creation of a network map based on text data, which reveals useful information for understanding the key topics attracting complete attention. Thirty-three selected papers have been included for the bibliometric analysis. Through a co-occurrence with a minimum of three, the software extracts 76 keywords (or items) from the full text of the selected papers. The keyword ‘shelter’, with a total link strength of 165, appeared as the most frequent keyword which had a strong link to ‘camp’, ‘refugee’ and ‘housing’. A network map was created to show the frequency of the terms which occurred more than five times. Figure 2 shows the network map, where each circle represents a keyword and the size of the circle represent the number of articles in which each keyword appears. In addition, the distance between the two terms represents the co-occurrence of the terms. A small distance between two terms represents a large number of co-occurrences.

As indicated in Fig. 2, there are six main clusters that represent different fields of research concerning health and shelter and their relations. The first cluster (red) focused on shelter environment and thermal comfort including terms such as ‘shelter’, ‘thermal comfort’, ‘thermal performance’ or ‘environment’. The second cluster (green) is focused on refugees and infectious diseases, with keywords terms ranked as ‘measles’, ‘risk factor’ and ‘outbreak’. The third cluster (blue) represents refugee camps and health. The main theme of the fourth cluster may be summarised as environmental health in displacement, while the fifth cluster (violet) may be summarized as housing design and refugee camps. Finally, the sixth cluster (turquoise) does not have a distinct focus, but the items with most co-occurrence are ‘earthquake’ and ‘household’. The largest cluster consisted of 26 items (red colour—shelter and thermal comfort) and it focuses on indoor thermal conditions in refugee shelters and shelter indoor environment. It is interesting to observe the high strength between ‘health’ and ‘air quality’, meaning that these topics are highly correlated, as well for items such as ‘ventilation’ and ‘indoor thermal environment’. It is observed that there are some items, such as 'vulnerability' and ‘recovery’ with low strength and lacking connection with the core of the cluster which is represented by shelter, refugee and camp.

### Systematic analysis

**Fig. 3:**
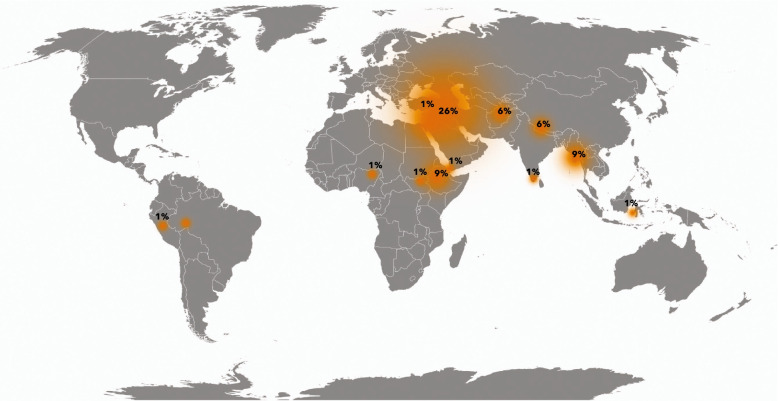
Percentage distribution of included papers over the world

**Fig. 4:**
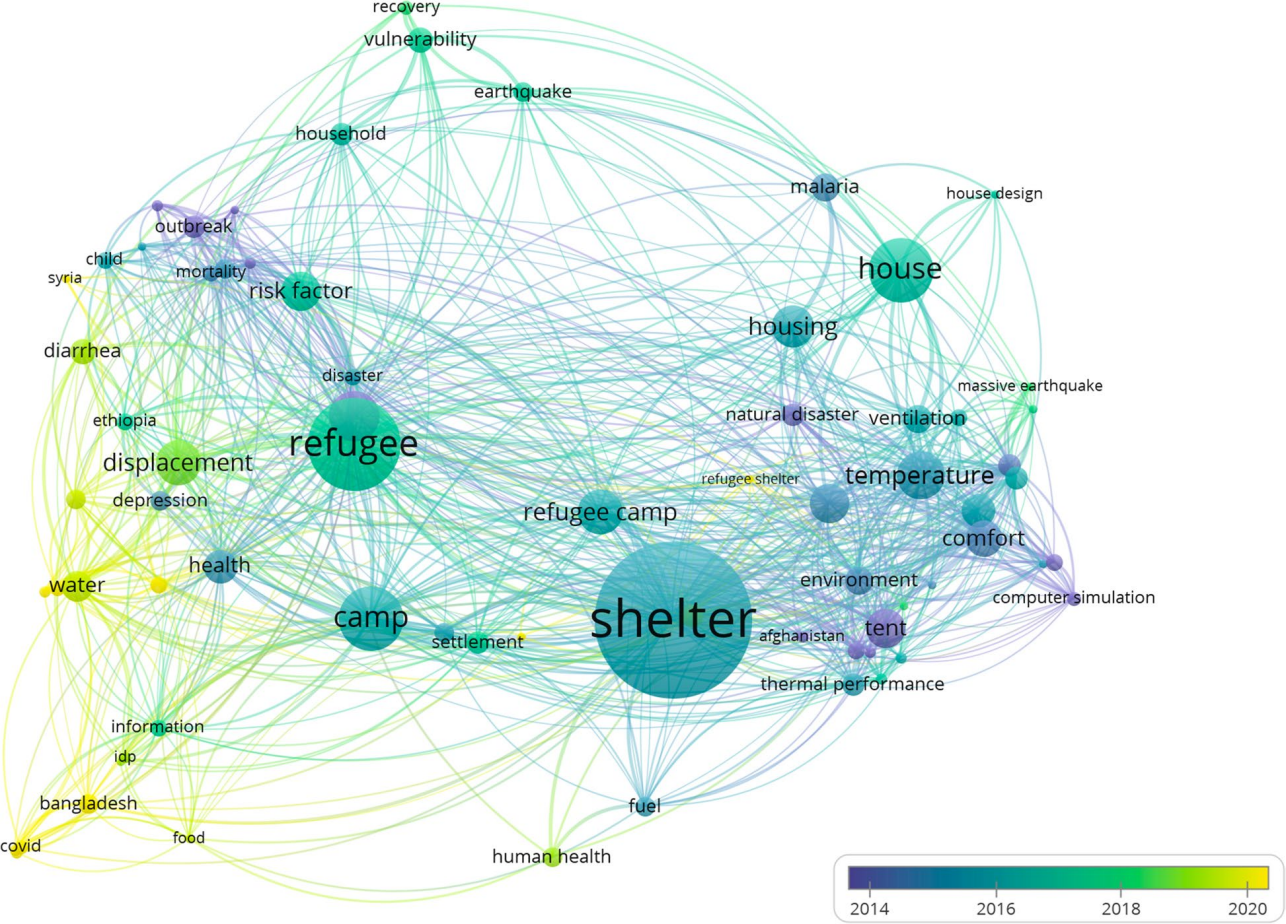
VOS viewer co-occurrence network visualisation mapping of the most frequent keywords in shelter and health over the years

The 33 selected papers have been published in the period 2000 to 2020, with an increase in papers published since 2016. Three papers are chapters in book, two are manuals and other two are reports. The remaining 26 are research papers. The description of the 33 research papers shows that Middle East attracts more attention, with 16 papers addressing this area. 14% of the studies focus on Africa, while only 1% focus on the South America Fig. 3. Most research have been conducted by academia in collaboration with NGOs and most of the research has been published mainly in 2018 as can be observed in Fig. 4. Not surprisingly Fig. 4 shows that recent developments in the field of shelter and health revolves around COVID-19 and airborne disease. Only five studies were designed with the purpose to explore the link between housing and health; Al-Betawi (Al-Betawi et al. 2020), Al-Khatib (Al-Khatib et al. 2003, 2005; Al-Khatib and Tabakhna 2006), Brevik (Brevik et al. 2019) and Klansek (Klansek et al. 2021). Most of the studies used interviews or semi-structured interviews or group interviews and field observations. The chapters in books (Cardozo and Mollica 2018; Marano and Ahmed 2018; Setchell et al. 2018) did not focus on shelter conditions in detail. However, all of them considered shelter as a risk factor of health and described the possible correlation between low shelter quality and health diseases. Table 1 summarizes the research papers included in the systematic analysis and describes which correlations between shelter issues and health have been found in the studies.

**Table 1:** Overview of the health diseases related to shelter issues in included journal papers (Literature reviews are not included)

Author	Year	Country	Type of disaster	Type of shelter	Shelter issues	Health related issue
Al-Betawi et al. 2020	2020	Jordan	Conflict	Temporary house	Overcrowded shelter	General poor health
Al-Khatib et al. 2003	2003	Palestine	Conflict	Temporary house	Overcrowded shelter Dampness Not adequate ventilation Not adequate sunlight Inadequate indoor temperature	Common cold (mostly in children)
Al-Khatib and Tabakhna 2006	2006	Palestine	Conflict	Temporary house	Overcrowded shelter Dampness Not adequate ventilation Not adequate sunlight Inadequate indoor. temperature	Common cold (mostly in children)
Albadra et al. 2017	2017	Jordan	Conflict	Temporary shelter	Inadequate indoor temperature	Thermal stress risk
Albadra et al. 2018	2018	Jordan	Conflict	Temporary shelter	Inadequate indoor temperature	Thermal stress risk
Albadra et al. 2020	2020	Bangladesh Djibouti Ethiopia Jordan Peru Turkey	Conflict and natural disaster	Temporary shelter	Inadequate indoor air	n.a
Araya et al 2011	2010	Ethiopia	Conflict	Temporary shelter	Inadequate shelter environment	Mental distress Reduced quality of life
Banik et al. 2020	2020	Bangladesh	Conflict	Temporary shelter	Overcrowded shelter	Vulnerability to coronavirus
Brevik et al. 2019	2019	n.a	n.a	n.a	Inadequate shelter environment	Vulnerability to extreme weather, animals, stress and health diseases
Chan et al. 2018	2018	Bangladesh	Conflict	Temporary shelter	Inadequate shelter materials	Increased exposure to vector-borne disease
Connolly et al. 2004	2004	n.a	n.a	Temporary shelter	Inadequate shelter environment Overcrowded shelter	Acuter respiratory infections Malaria
Cornaro et al. 2015	2015	n.a	n.a	Emergency shelter	Inadequate indoor temperature	n.a
Crawford et al. 2005	2005	n.a	n.a	Emergency shelter	Inadequate indoor temperature	n.a
Dominguez-Amarillo et al. 2021	2021	Afghanistan Jordan South Sudan	Conflict	Temporary shelter	Inadequate indoor temperature	Thermal stress risk
Ekezie et al. 2019	2018	Nigeria	Conflict	Temporary shelter	Inadequate shelter environment Overcrowded shelter	Health risk
Fosas et al. 2017	2017	Jordan	Conflict	Temporary shelter	Inadequate indoor temperature	Thermal stress risk
Fosas et al. 2018	2018	Jordan	Conflict	Temporary shelter	Inadequate indoor temperature	Thermal stress risk
Hamdan et al. 2021	2021	Jordan	Conflict	Tent	Inadequate indoor temperature	Thermal stress risk
Hammer et al. 2018	2018	n.a	n.a	n.a	Inadequate shelter environment Overcrowded shelter	Health risk
He et al. 2018	2018	Nepal	Natural disaster	Temporary shelter	Inadequate shelter materials	Health risk
IRC 2020	2020	n.a	n.a	n.a	Inadequate shelter environment Overcrowded shelter	Exposure to disease transmission
Klansek et al. 2020	2020	Bangladesh	Conflict	Temporary shelter	Inadequate shelter materials Inadequate indoor air Inadequate indoor temperature Overcrowded shelter	Health risk
Kouadio et al. 2010	2010	n.a	n.a	n.a	Overcrowded shelter	Measles transmission
Manfield et al. 2004	2004	n.a	n.a	n.a	Inadequate indoor temperature	Health risk
Fosas et al. 2020	2021	Jordan	Conflict	Temporary shelter	Inadequate indoor temperature	Thermal stress risk
Obyn et al. 2015	2015	n.a	n.a	Emergency shelter	Inadequate indoor temperature	Thermal stress risk
Saleh et al. 2012	2012	Palestine	Conflict	Temporary shelter	Inadequate indoor temperature	Thermal stress risk
Shackelford et al. 2020	2020	n.a	n.a	Emergency shelter	Inadequate shelter environment	Health risk
Susanti, 2015	2015	Indonesia	Natural disaster	Emergency shelter	Inadequate indoor temperature	Thermal stress risk
Thapa et al. 2018	2018	Nepal	Natural disaster	Temporary shelter	Inadequate indoor temperature	Thermal stress risk
Tuladhar et al. 2019	2020	n.a	n.a	Temporary shelter	Inadequate indoor temperature	Thermal stress risk
Turner et al. 2009	2009	Sri Lanka	Natural disaster	Temporary shelter	Inadequate shelter environment Overcrowded shelter	Risk factor for cough, stomach ache, headache
Zabaneh, 2008	2008	Lebanon	Conflict	Temporary house		

## Comparative analysis

Poor shelter conditions are associated with a wide range of communicable, non-communicable and mental health diseases (Ziersch and Due 2018), as reported by the last WHO report (WHO 2018). Communicable diseases are the primary cause of death in a displaced population in low-income countries (Hammer et al. 2018). Often, these deaths occur in the emergency phase of a disaster (Culver et al. 2017) and children under five have the worst mortality rate (Dubray and Guha-Sapir 2018). Common communicable diseases in an emergency include diarrheal disease and cholera, infection, meningitis, measles, malaria and respiratory diseases. Not only overcrowded shelters are considered one of the most influential factors in the spread of communicable diseases (Turner et al. 2009; Alnsour and Meaton 2014), but also the presence of mould and damp due to poor air quality can contribute to the incidence of communicable diseases among refugee populations. Physical diseases associated with mental health are also increasing and the impact of mental illness is considered more significant amongst displaced populations (Cardozo and Mollica 2018) than in the rest of society. According to Feyera (Feyera et al. 2015), lack of adequate shelter is significantly associated with depression. Similar outcomes were also found by Carta (2013) in a cross-sectional survey in Burkina Faso and by Akinyemi (Akinyemi et al. 2012, 2016) in a Nigerian refugee camp.

**Fig. 5:**
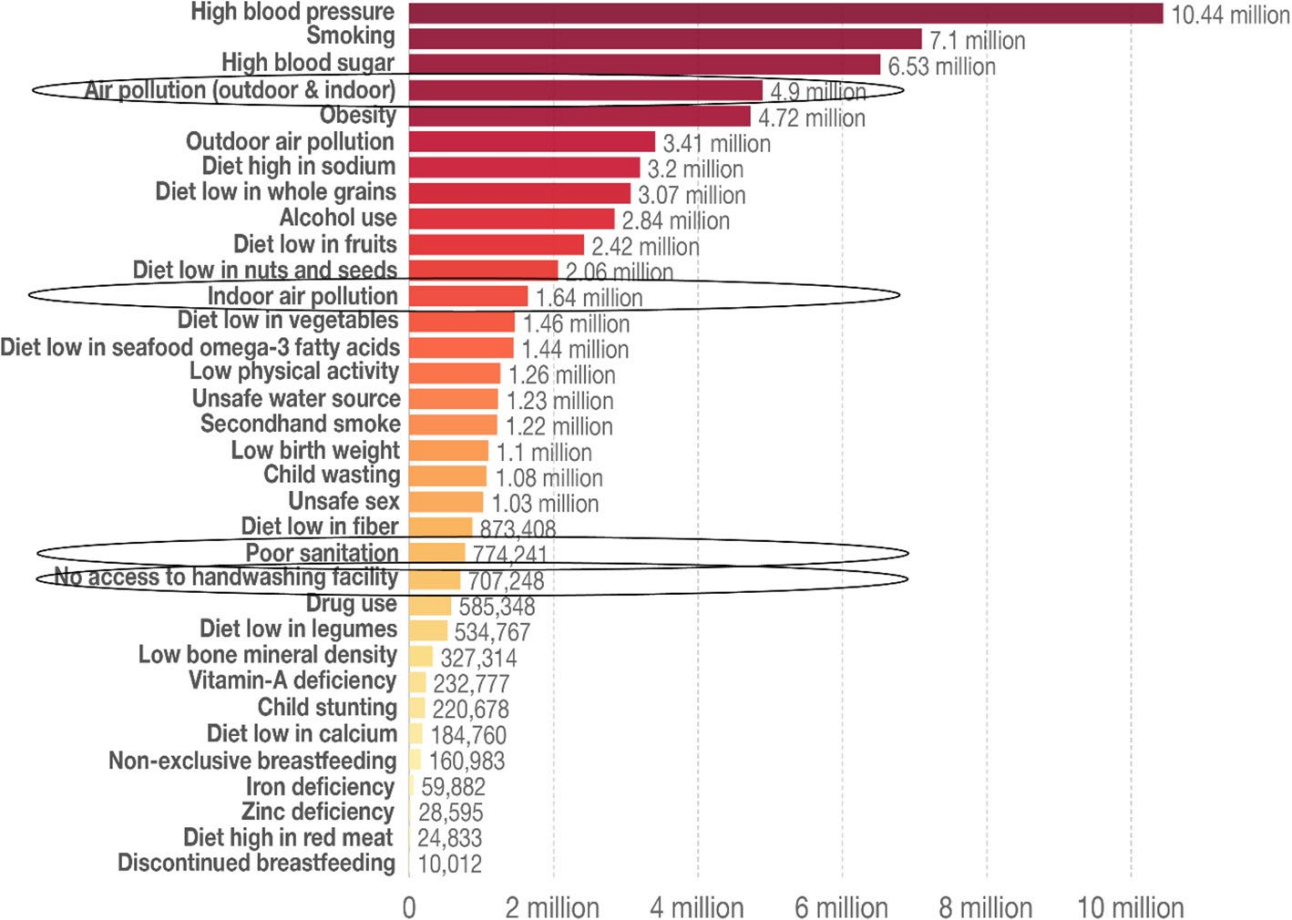
Number of deaths by risk factors, 2017. Adapted from Global Burden of Disease Database

Living conditions, including indoor air pollution and housing quality, are associated with global deaths, as shown in Fig. 5. Housing affects health through a series of risk factors including indoor air pollution, overcrowding, thermal extremes and damp and mould. In low-income countries and in post-disaster and post-conflict scenarios, the impact of these risk factors is higher than for the global population.

This section, summarised in Table 2, describes the studies included in the comparative analysis, focusing on the most frequent shelter issuer related to human health.

**Table 2:** Comparative analysis of selected studies regarding thermal comfort and indoor air quality assessment and their findings

				**Method**					**Findings**	
**Research**	**Year**	**Shelter type**	**Location**	**Monitoring**	**Simulation**	**Thermal comfort surveys**	**Social survey**	**Evaluation**	**Thermal comfort/air quality**	**Health**
Manfield, 2004	(2004)	Tent	Kosovo	•	•			Design	New insulated roofing solution performed better than the UNHCR winterized tent	–
Crawford, 2005	(2005)	Tent	Islamabad London Pristina		•			Design	UNHCR tent was unable to maintain heath inside without a heating source	–
Saleh, 2012	(2012)	Temporary shelter	Palestine		•	•		Shelter in use	Statistically significant difference between TSV and PMV	Extremely uncomfortable thermal conditions in summer and in winter
Cornaro, 2015	(2015)	Tent	Torino Palermo		•			Design	Insulation decreases the number of hours of cold discomfort but increases those of heath discomfort. The solution with insulation and shading offers the most acceptable conditions	–
Obyn, 2015	(2015)	Tent	Belgium Burkina Faso Luxembourg		•			Design	Overcooling during night and ground temperature were difficult to model	–
Susanti, 2015	(2015)	Tent	Indonesia	•		•		Shelter in use	Air temperature and velocity did not meet the standards ASHRAE-55 and ISO 7730	Households were satisfied of their thermal environment
Thapa, 2016	(2016)	Temporary shelter	Nepal	•		•		Shelter in use	Low levels of thermal comfort were registered in autumn	–
Albadra, 2017	(2017)	T-shelter	Jordan	•	•	•	•	Shelter in use	High temperatures were registered in shelter. PMV underestimated the adaptive potential of the refugees	–
Fosas, 2017	(2017)	T-shelter	Jordan	•	•	•	•	Shelter in use	High surface temperatures. Unsatisfying thermal conditions especially in summer	Unsatisfying living conditions, especially in summer
Thapa, 2018	(2018)	Temporary shelters	Nepal	•		•		Shelter in use	Extreme indoor temperatures in summer and winter. Comfort temperature was highly correlated with the outdoor air temperature	Uncomfortable indoor temperatures
Wang 2019	(2019)	PHT	China		•			Design	Adding phase change materials to all shelter surface can improve indoor thermal conditions	–
Tuladhar, 2020	(2020)	IFRC shelter	14 different location		•			Design	Indoor temperature in all simulated shelters exceeded the adaptive thermal comfort threshold	–
Klansek, 2020	(2020)	Temporary shelter	Bangladesh	•		•	•	Shelter in use		–
Albadra	(2021)	Temporary shelter		•				Shelter in use	Indoor shelter conditions were characterized by insufficient ventilation, poor air quality and low thermal comfort	–
Hamdan, 2021	(2021)	Tent			•			Design	Natural ventilation and roof insulation can enhance the thermal performances of the UNHCR tent	–

### Overcrowding

Overcrowding measures the relationship between the number of persons and the shelter space capacity. It is defined as an environmental determinant of health and well-being and it is often associated with poor health outcomes. Shaw was the first to observe a link between overcrowding and various health outcomes (Shaw 2004) and all the shelter guidelines since have adopted minimum standards for shelter space per person (Sphere Project 2018). Taioli (Taioli et al. 2018) reported that chaotic and poorly organised shelter were perceived as more traumatic by households. Participants identified in overcrowded and uncomfortable shelter a determinant of both physical and mental health. Al-Betawi (Al-Betawi et al. 2020) observed that refugees living in overcrowded shelters are more at risk of general poor health than displaced families living in houses in an urban context. Zabaneh (2008), by studying the living conditions of Palestinian refugees in an unofficial camp in Lebanon, found that households who lived in a prolonged overcrowded environment were more susceptible to develop chronic diseases. Al-Khatib (Al-Khatib and Tabakhna 2006) identified overcrowding as one of the causes of respiratory conditions among inhabitants of Jalazone Refugee Camp and reported a statistically significant relationship between overcrowding and upper respiratory tract infections and ear infections. Overcrowded shelters also increased the frequency of diarrheal diseases such as cholera and gastrointestinal diseases (Tabbaa and Seimenis 2013) and the risk for displaced people to contract malaria (Tusting et al. 2020; Jatta et al. 2018; Williams et al. 2018; Furnival-Adams et al. 2019). In addition, the high density of households inside shelters is directly associated with measles outbreaks and risk of cough in the displaced population (Connolly et al., 2004; Kouadio et al. 2010), as found in Tanzanian refugee camps by Turner (Turner et al. 2009). The combination of poor hygiene and overcrowding contributes to the spread of skin infection such as scabies and pediculosis (Fazel et al. 2014). More recently, Al-Betawi (Al-Betawi et al. 2020) found a correlation between the high number of people per room and general poor health in displaced persons in Jordan. In addition, recent studies on COVID-19 (Banik et al. 2020), included overcrowded shelters among the risk factors for the spread of the virus among Rohingya refugees in Bangladesh.

### Air quality

Displaced persons are often encamped in single or two room shelters, where all the occupant’s activities such as sleeping, cooking or socialising take place. Usually, these shelters are not provided with a proper system of ventilation or flues for smoke (Buu et al. 2014). This results in inadequate levels of IAQ due to the high presence of volatile organic compounds (VOCs), particulate matter (PM), and carbon dioxide (CO_2_). High levels of PM have been associated with respiratory and cardiovascular diseases, while microbial agents encourage the development of asthma and allergies. Low air quality, associated with insufficient ventilation encourages mould growth and the lack of adequate ventilation encourages the spread of respiratory infections such as colds and coughs, especially among children, as reported by Al-Khatib (Al-Khatib and Tabakhna 2006). According to Marano and Ahmed (Marano and Ahmed 2018), insufficient ventilation in shelters combined with indoor air pollution increases the incidence and transmission of acute respiratory infections such as tuberculosis. This is particularly evident in warm climates, when windows may be closed to maintain thermal comfort and security (Nardell 2016) or to preserve privacy (Obyn et al. 2015; Albadra et al. 2017; Asgary and Azimi 2019) especially for women (Al-Khatib et al. 2005).‬‬‬‬‬‬‬‬‬‬‬‬‬‬‬‬‬‬‬‬‬‬‬‬‬‬‬‬‬‬‬‬‬‬‬‬‬‬‬‬‬‬‬‬‬‬‬‬‬‬‬‬‬‬‬‬‬‬‬‬‬‬‬‬‬‬‬‬‬‬‬‬‬‬‬‬‬‬‬‬‬‬‬‬‬‬‬‬‬‬‬‬‬‬‬‬‬‬‬‬‬‬‬‬‬‬‬‬‬‬ Inadequate airflow is also indirectly related to the spread of malaria (Kouadio et al. 2010). For example, Von Seidlein (2012) reported an attenuation of the airflow due to the use of ITN (Insecticide-treated nets) and a consequent increase in the level of thermal discomfort, particularly during the hottest months of the year. The authors also observed that this time coincides with the peak of malaria infections and households preferring not to use bed nets are exposing themselves to a significant risk of contracting malaria.‬‬‬‬‬‬‬‬‬‬‬‬‬‬‬‬‬‬‬‬‬‬‬‬‬‬‬‬‬‬‬‬‬‬‬‬‬‬‬‬‬‬‬‬‬‬‬‬‬‬‬‬‬‬‬‬‬‬‬‬‬‬‬‬‬‬‬‬‬‬‬‬‬‬‬‬‬‬‬‬‬‬‬‬‬‬‬‬‬‬‬‬‬‬‬‬‬‬‬‬‬‬‬‬‬‬‬‬‬‬‬‬‬‬‬‬‬‬‬‬‬‬‬‬‬‬‬‬‬‬‬‬‬‬‬‬‬‬‬‬‬‬‬‬‬‬‬‬‬‬‬‬‬‬‬‬‬‬‬‬‬‬‬‬‬‬‬‬‬‬‬‬‬‬‬‬‬‬‬‬‬‬‬‬‬‬‬‬‬‬‬‬‬‬‬‬‬‬‬‬‬‬‬‬‬‬‬‬‬‬‬‬‬‬‬‬‬‬‬‬‬‬‬‬‬‬‬‬‬‬‬‬‬‬‬‬‬‬‬‬‬‬‬‬‬‬‬‬‬‬‬‬‬‬‬‬‬‬‬‬‬‬‬‬‬‬‬‬‬‬‬‬‬‬‬‬‬‬‬‬‬‬‬‬‬‬‬‬‬‬‬‬‬‬‬‬‬‬‬‬‬‬‬‬‬‬‬‬‬‬‬‬‬‬‬‬‬‬‬‬‬‬‬‬‬‬‬‬ As mentioned before, insufficient airflow is also associated with damp and mould that are in turn linked to several health diseases, including respiratory infections and asthma (Checchi et al. 2007; WHO 2014).

Poor levels of IAQ are also strongly connected with presence of damp and mould, which act to pollute indoor air with VOCs. The presence of mould and damp varies among climatic zones and it is more severe in developing countries. Persistency of dampness and mould promote microbial growth and survival of viruses which can transmit infections and allergens. In a study among refugees in Alma’ri refugee camp in Palestine (Al-Khatib et al. 2003), 72% of the homes were found to have damp or mould and according to the participants this was the cause of most of their infections. Similar outcomes were found by the same author in Jalazone refugee camp (Al-Khatib and Tabakhna 2006). Refugees self-reported a correlation between living in houses with common mould and symptoms of common cough and asthma. In Zabaneh (2008), Palestinian refugees reported the presence of mould and damp especially in unheated houses and in houses with no external ventilation. Such conditions were correlated with poor health, particularly, with respiratory diseases.

### Indoor temperatures

The majority of displaced populations are encamped in areas with extreme climates. In countries like Syria, Jordan and Lebanon, the outdoor temperatures during winter can drop below zero. As such, the lightweight materials used in shelter construction, often do not provide an adequate thermal environment and indoor temperatures may be extremely low (UNHCR 2022). Cold indoor temperatures may result in airborne infection, respiratory diseases and stress to the cardiocirculatory system. In the last 5 years, several studies have provided growing evidence for the impact of indoor cold temperatures (World Health Organization 2018b) and how insulation can improve health outcomes.

While growing evidence exists in support of the impact of cold indoor temperature, less evidence exists showing the link between high indoor temperatures and health. However, the attention on the effects of high temperatures on health is increasing, particularly because of climate change. High outdoor temperatures result in high indoor temperatures, especially in displacement conditions, where people do not have access to air conditioning, insulation, specific building materials or they cannot exploit natural ventilation due to cultural aspects, security or privacy. High indoor temperatures are associated with thermal discomfort, and recently the interest in its effects on health has increased due to climate change and the frequency of heatwaves. Prolonged exposure to extreme warm results in increased morbidity and mortality especially among vulnerable populations (Lee et al. 2016).

The Healthy Housing for the Displaced project (Albadra et al. 2018) carried out research on shelter indoor environment in different locations. Empirical studies in Jordan (Albadra et al. 2017), Ethiopia (Paszkiewicz and Fosas 2019) and Bangladesh, demonstrate that measured air temperatures inside shelters is often greater than 40 °C. This represents a potential indoor heath stress as estimated by Steadman’s Apparent Temperature (Steadman 1979, 1984) for indoor environments. In Arzaq refugee camp Fosas (Fosas et al. 2017) demonstrated that refugees are subject to overheating for the 20% of the year. However, the study also observed that through a series of retrofit adaptations it is possible to reduce the effects of excess heat on health.

Over the last 5 years, a growing number of authors have recognised the importance of the thermal performance of shelters in both cold and hot climates. Attia (Attia 2014) assessed the thermal performance of Bedouin tents in a warm climate intending to improve thermal comfort without compromising the design. The study highlighted the importance to conduct structured interviews among the population to understand the use of the indoor space. The author, however, did not investigate the link between health and thermal comfort. Thapa (Thapa et al. 2018, 2019) conducted a field survey in the aftermath of the Nepalese earthquake of 2015 to monitor the seasonal changes of indoor environment and to find an acceptable temperature range in self-built shelters. The authors found that the comfort temperatures vary following the globe temperature’s variations and that are strongly related to the indoor temperature. Benefits of improved natural ventilation have been described in a recent study by Hamdan (Hamdan et al. 2021). The author simulated the thermal behaviour of the UNHCR emergency tent, combining different passive strategies. However, the paper did not mention possible implications with human health correlated to thermal comfort or ventilation.

## Investigation of shelter design factors

The analysed studies mentioned three main shelter environment factors having an impact on the health of the displaced, such as overcrowding, indoor temperatures and ventilation.

Overcrowding resulted to be an environmental factor associate with shelter design and stogly linked to the health of shelter occupants. However, it has to be clarified that not always overcrowding is a result of inappropriate shelter design.

The impact of overcrowding in shelters is mentioned in several studies, nevertheless, it has never been measured or objectively assessed. None of the included studies mentioned the Sphere minimum standard of 3.5 m^2^ per person when they analyse the impact of overcrowded shelters on occupants health; therefore, an objective assessment for overcrowding does not exist.

Instead, three distinct methodologies exist for assessing the impact of IAQ and indoor temperatures on refugee and IDPs' health: objective approach (on-field measurements), subjective approach (surveys) and dynamic thermal model simulations. The objective approach is based on field measurements campaigns, while the subjective approach is conducted by giving refugees and IDPs a survey about their personal perceived level of comfort. Dynamic thermal modelling simulation can be used to predict the risk of overheating in shelters, by calculating the heat balance of the shelter consecutively for each time step and the concentration of pollutants of the indoor air.

Field measurements and observation are largely used due to the peculiar factors affecting thermal comfort and IAQ such as climate, remoteness of the site, culture, political and security requirements, design and physical and emotional status. However, conducting on-site research in post-disaster and post-conflict scenarios may be challenging due to the unpredictability of the scenario and remoteness of the site. In addition, the accuracy of the measurements is subject to the quality of the instruments and the accuracy of the used sensors.

### Investigation of IAQ

IAQ assessment is determined by a wide range of factors such as levels of VCOs, PM or CO_2_. But the identification of the hazard needs to include a variety of cultural habits and way of living that vary across the world. Presence of chemicals that affect health is obtained from toxicological databases. However, effects of exposure are not available for some of these chemicals. In developed countries, there are several environmental assessment schemes and building regulations which provide guidelines for IAQ such as UK Air Quality Strategy. However, for developing countries the guidance on recommended levels of IAQ is limited. WHO provides some guidelines on the harmful level of PM, and pollutants levels. Others environmental assessment schemes, such as BREEAM (2021) and LEED (2008) offer guidance on air quality, but there is not any standard referring to shelter. Existing studies, using interviews with households, found a correlation with the presence of health diseases (Moffa et al. 2019; Behnke et al. 2020) such as airborne infections (Marano and Ahmed 2018). Recently, Albadra (Albadra et al. 2020) conducted a cross country study on the measure and analysis of air quality in temporary shelters. The authors monitored shelter using spot measurements. Despite the challenges of fieldwork that did not allow a 24 h monitoring, they demonstrated that high levels of CO_2_, PM and VOCs determine low air quality.

### Investigation of indoor temperatures

Dynamic thermal simulation can be used to perform the thermal behaviour analysis considering construction materials, internal gains, occupancy parameters and natural ventilation. The thermal model analyses the annual internal shelter temperatures based on the climatic conditions of temperature and humidity. This methodology allows the study of most of the parameters related to the shelter environment. It is time effective and low cost (Fosas et al. 2020) and allows to capture the indoor behaviour of the shelter.

Most of the reviewed studies (Manfield et al. 2004; Crawford et al. 2005; Obyn et al. 2015) concerning shelter thermal comfort relied on building simulation to evaluate shelter thermal performances. Ajam (1998) conducted one of the first studies using shelter modelling to improve thermal comfort in Jordanian refugee shelters. The author observed that roof insulation and night ventilation played a key role in improving thermal comfort. Manfield (Manfield et al. 2004) used shelter simulation to compare alternative roofing material for mitigating the effects of cold climate. Results showed that the prototyped shelters, with new roofing solutions, performed better than the UNHCR winterized tent. Other authors applied building simulation to study thermal performances of emergency tents in cold climates. Crawford (Crawford et al. 2005) simulated two different shelters in selected locations in the world, Islamabad, London and Pristina. Authors observed that unheated tents had the greatest problems with humidity and underventilation. Cornaro (Cornaro et al. 2015) modelled a tent using dynamic simulation in two different climatic conditions. The winter case was simulated using climatic data from Torino and the summer case using climatic data from Palermo. The authors simulated two improved solutions for the winter case and four for the summer case. They observed that the best conditions were offered by the improved solution with both insulation and shading. Similarly, Obyn (Obyn et al. 2015) used dynamic simulation to model a standard IFRC tent and then the authors calibrated the model using field data collected in Belgium, Burkina Faso and Luxembourg. The authors found that some phenomena such as overcooling during night and ground temperature were difficult to model. Also, they underlined the wide impact of the ground temperature on results. Recently Tuladhar (Tuladhar et al. 2019) used thermal simulation to investigate thermal-safety in fourteen most common temporary shelters in their originally intended location. The authors created the model of each shelter following the IFRC catalogue. The results showed that indoor temperature in shelter exceeded the adaptive thermal comfort threshold ASHRAE-55 (2017). The authors observed also that simulation can be more effective when using shelters due to their simplicity. The author also demonstrated that dynamic simulation applied to shelters is ideal for testing alternative materials and design strategies.

Saleh (Saleh and Gadi 2012) employed both questionnaires and dynamic simulation to investigate shelters in refugee camps in Palestine. The authors considered the predicted mean vote (PMV) estimated by computer simulation and the thermal sensation vote (TSV) gathered by questionnaires to study thermal environment in shelters in Jabalia refugee camp in Palestine. In addition, they answered questions on air humidity, air circulation and indoor solar radiation. Both methods found extremely uncomfortable thermal conditions in summer and in winter. It was also observed a statistically significant difference between TSV and PMV. However, the mean of PMV-TSV discrepancies was acceptable. Therefore, dynamic simulation was considered a reliable method to predict thermal comfort.

Thapa deeply investigated the thermal conditions of temporary shelters in Nepal. In a preliminary study (Thapa et al. 2016) conducted in the aftermath of the 2015 earthquake in Nepal, the author evaluated the thermal environment in temporary shelter and the thermal comfort of habitants. The study included indoor thermal measurements and questionnaires based on thermal comfort surveys. Results found low levels of thermal comfort especially in autumn and described different adaptation used by occupants to maintain thermal comfort. From the same author, a series of surveys was conducted in Nepal to assess the acceptable indoor temperature in temporary shelters (Thapa et al. 2018) to estimate the mean radiant temperature, the comfort temperature and the thermal acceptability. They found a positive correlation between indoor air temperature and outdoor temperature. They also observed that displaced people tried to maintain thermal comfort by using personal techniques and retrofits. Susanti (Susanti 2015) evaluated thermal comfort of emergency tents in Indonesia. The authors predicted PMV and PPD (predicted percentage of dissatisfied) measuring environmental parameters and information from questionnaire and observations. Results showed that household were satisfied of their thermal environment although air temperature and air velocity did not respect the limits of the two selected standards ASHRAE-55 and ISO 7730.

Albadra (Albadra et al. 2017) for the first time studied thermal conditions in Azraq and Zaatari refugee camp in Jordan through the use of thermal comfort surveys and physical measurements. The authors combined social and thermal comfort surveys and physical measurements to assess the environmental conditions in shelters and to discover common thermal adaptation methods. Authors observed that the comfort band ranged from 28.4 °C to 17.2 °C. This suggested that displaced persons have significant adaptation capacity. They also found that PMV model is not a reliable model for use in extreme environments. Fosas (Fosas et al. 2017) used surveys, monitoring of indoor shelter conditions and building and human thermal simulation to evaluate the annual overheating risk of refugees. Results showed that increasing internal mass is positive associated with a reduction of heat stress. Recently, in a study conducted in Bangladesh by Klansek (2020) on-site measurements, focus group discussions, semi structured interviews and large-scale assessment were conducted to analysed not only thermal conditions inside shelters, but also to obtain a more holistic description of shelter issues. Results reported insufficient ventilation, poor air quality and low thermal comfort, demonstrating the benefits of using a mixed-method approach to assess thermal comfort.

## Discussion

The assessment of the impact of shelter on health involves the analysis of several parameters and different methodologies including interviews, building modelling, field measures and health data analysis and different stakeholders such as humanitarian actors, researchers and displaced population. Generally speaking, studies on shelter and health are hard to compare since they are highly site-specific, due to the peculiarities of each post-disaster and post-conflict scenario the heterogeneity of the displaced population and the characteristic of each shelter.

### Results of bibliometric and systematic analysis

The systematic bibliometric analysis of the literature was not particularly fruitful. Out of 35 papers meeting the inclusion criteria, only 4 papers were specifically designed to directly assess the impact of shelters on occupants’ health (Al-Khatib et al. 2003, 2005; Al-Khatib and Tabakhna 2006; Zabaneh et al. 2008). The literature linking shelter and health is very scant and the available studies only gave some recommendations about healthy shelter design, without conducting data analysis to prove the link. The bibliometric analysis demonstrated that the relationship between shelter and health included two main fields of research: shelter design, and the health of displaced populations. The several overlaps showed that there is not yet a clear path of research analysing specifical aspects of the impact of shelter on occupants' health. The theme of healthy shelter in displacement is still overlooked and defined methodologies have never been applied to assess the impact of shelter on human health.‬‬‬‬‬‬‬‬‬‬‬‬‬‬‬‬‬‬‬‬‬‬‬‬‬‬‬‬‬‬‬‬‬‬‬‬‬‬‬‬‬‬‬‬‬‬‬‬‬‬‬‬‬‬‬‬‬‬‬‬‬‬‬‬‬‬‬‬‬‬‬‬‬‬‬‬‬‬‬‬‬‬‬‬‬‬‬‬‬‬‬‬‬‬‬‬‬‬‬‬‬‬‬‬‬‬‬‬‬‬‬‬‬‬‬‬‬‬‬‬‬‬‬‬‬‬‬‬‬‬‬‬‬‬‬‬‬‬‬‬‬‬‬‬‬‬‬‬‬‬‬‬‬‬‬‬‬‬‬‬‬‬‬‬‬‬‬‬‬‬‬‬‬‬‬‬‬‬‬‬‬‬‬‬‬‬‬‬‬‬‬‬‬‬‬‬‬‬‬‬‬‬‬‬‬‬‬‬‬‬‬‬‬‬‬‬‬‬‬‬‬‬‬‬‬‬‬‬‬‬‬‬‬‬‬‬‬‬‬‬‬‬‬‬‬‬‬‬‬‬‬‬‬‬‬‬‬‬‬‬‬‬‬‬‬‬‬‬‬‬‬‬‬‬‬‬‬‬‬‬‬‬‬‬‬‬‬‬‬‬‬‬‬‬‬‬‬‬‬‬‬‬‬‬‬‬‬‬‬‬‬‬‬‬‬‬‬‬‬‬‬‬‬‬‬‬‬‬‬‬‬‬‬‬‬‬‬‬‬‬‬‬‬‬‬‬‬‬‬‬‬‬‬‬‬‬‬‬‬‬‬‬‬‬‬‬‬‬‬‬‬‬‬‬‬‬‬‬‬‬‬‬‬‬‬‬‬‬‬‬‬‬‬‬‬‬‬‬‬‬‬‬‬‬‬‬‬‬‬‬‬‬‬‬‬‬‬‬‬‬‬‬‬‬‬‬‬‬‬‬‬‬‬‬‬‬‬‬‬‬‬‬‬‬‬‬‬‬‬‬‬‬‬‬‬‬‬‬‬‬‬‬‬‬‬‬‬‬‬‬‬‬‬‬‬‬‬‬‬‬‬

None of the reviewed studies investigating thermal comfort or IAQ in shelters had also measured the health implications. Recently, several authors investigated the thermal comfort in shelter, founding that indoor temperatures reach high levels, especially in summer, jeopardising the life of occupants. This observation descends from a comparison with standards such as ASHRAE-55 or ISO 7730 that usually do not refer to shelters. IAQ was rarely analysed. As a unique example, Albadra (Albadra et al. 2020) conducted a cross-country study measuring IAQ in refugee camps and temporary shelters. This is surprising because IAQ is considered among the main determinants of human health and is one of the risk factors for deaths worldwide. In addition, levels of IAQ in shelter should be of particular attention. Often, in fact, cooking occurs inside shelters using fossil fuels and health issues related to these habits were reported. Even rarely IAQ and thermal comfort were analysed or simulated together even though they are interdependent.

### Results of comparative analysis

The comparative analysis presented here has proven useful in answering the research questions. Analysed papers do not mention any of the existing guidelines for shelter design present in the manuals used for the preliminary reading such as the Sphere Project. From the analysis of the included papers, the main factors of shelter design affecting human health are overcrowding, uncomfortable indoor temperature and poor indoor air quality as explained in Table 3. Clear guidelines exist for overcrowding and they refer to the minimum square metreage per person for designing the shelter area. For thermal comfort and indoor air quality, there are suggested design strategies to enhance the indoor environment of a shelter, but the guidelines do not provide a threshold or a standard in terms of healthy temperatures, airflow or indoor concentration of pollutants. Without a clear standard, it is challenging to find the right strategy for improving thermal comfort and indoor air quality for shelters.

**Table 3:** Shelter related issues and their impact on the health of occupants

Shelter indoor environment issue	Potential shelter design issue	Potential health issue
Dampness and mould^a^	Lack of adequate materials (especially insulation) Lack of adequate ventilation Low indoor temperatures	Asthma Common cold and cough Colds Eye irritation Possible respiratory infection
High indoor temperatures	Shelter material Shelter construction Use of open stove	Health issues are not well defined as the exposure to high indoor temperatures has never been investigated. Even if there is no direct evidence on the health outcomes associated with high indoor temperatures, these are linked to increase of mortality and issue to the cardiovascular and immune system
Lack of adequate ventilation	Small window dimension, wrong ventilation strategy	Eye irritation Airborne infections
Low indoor temperatures	Shelter material Shelter construction	Asthma Increased respiratory morbidity High blood pressure
Overcrowding	Inadequate shelter dimension Inadequate relationship between number of occupants and floor area	Infectious diseases: Respiratory infection Tuberculosis Diarrheal diseases Poor mental health Sleep deprivation
Poor indoor air quality	Small window dimension, wrong ventilation strategy. Presence of open stove in the shelter	Allergies Eye and skin irritation Airborne diseases Cardiovascular diseases

^a^Dampness and mould have been inserted as an independent factor in this table. However, their presence may be strongly related to the indoor temperatures and the indoor air quality and ventilation

It is important to highlight also, that overcrowding, uncomfortable indoor temperatures and poor indoor air quality are only mentioned as possible factors having an impact on health. None of the studies included in this review has proven or measured the impact of these design factors on the health of the displaced living in shelters. For example, studies measuring the indoor concentration of pollutants hypothesised that indoor air quality may increase morbidity and mortality due to respiratory disease. Also, they only hypothesised that shelter ventilation may be directly related to poor indoor air quality. However, no measure of shelter airflow has been provided as evidence of the link with high indoor concentration of pollutants and consequently the related health issues.

The comparative analysis shows that among the environmental factors of shelters, only indoor temperature and indoor concentration of pollutant have been measured and only indoor temperatures have been assessed for thermal comfort.

Additionally, from the records obtained for indoor temperatures and indoor air quality, it is still unclear how shelters determine the development of certain diseases. For instance, there is evidence that poor air quality increases the incidence of respiratory disease and that overcrowding is linked to respiratory infections and diarrheal disease. However, none of the studies provides a measure of this relationship.

### Thermal comfort assessment

Often, displacement camps are situated in remote areas with extreme climatic conditions. However, due to the unpredictability of the emergency scenario and to economic and political considerations, currently available shelters have been designed to be lightweight, low cost, and dismountable, with less attention paid to their thermal performance. Existing shelters fail to provide an acceptable indoor environment and protection against harsh temperatures. It is therefore surprising that there are only a few studies underlining the impact of indoor high temperatures on refugees’ health. Although the importance of thermal performance in shelter design it is widely known (Zetter 2012; Fosas et al. 2017), existing literature offers very few examples of the impact of exposure to indoor heat on displaced health. Albadra and Fosas (Albadra et al. 2017; Fosas et al. 2019) have deeply investigated the effects of high indoor temperatures inside shelters, underlining the unhealthy indoor temperatures and Albadra established an acceptable temperature for summer comfort. Assessing the impact of thermal comfort on human health remains challenging, due to the lack of guidelines linking shelter design and thermal comfort and the impossibility to apply western thermal standards and regulations in areas with extreme climates. Additionally, investigating thermal comfort is difficult as it involves several environmental parameters, human sensations and perceptions. The evaluation of thermal comfort has been conducted through three different approaches. However, each of them presents some drawbacks. Field measurements depend on the accuracy of the sensor and often, in refugee camps it is only possible to obtain spot measurements. Surveys and self-assessments may be misleading and the collaborations with refugees and IDPs need to be carefully evaluated due to ethical issues. Physics-based building simulation relies on the quality of the input data, which makes predicting thermal comfort in shelters challenging. Shelters are often self-built and built to unknown qualities and some parameters such as the U-values of walls, internal gains and ventilation strategies are difficult to obtain. Additionally, mass-produced shelters present some uncertainties because the occupants perform retrofits adding new unknown materials and techniques. Thermal modelling simulation is often based on default assumptions and borrowed templates and methodologies. This results in models that do not necessarily represent the cultural context and local practices or households’ adaptations. This suggests the interdisciplinary use of these three methods as a potentially reliable alternative to evaluate the impact of thermal comfort on health. However, as observed from the comparative analysis, dynamic simulation remains a more effective tool if applied for shelters because of their simpler nature in terms of design and lack of mechanical systems. In addition, testing new materials and design alternatives to improve thermal comfort may be simpler with thermal simulation. With the use of simulation, it is possible to simulate the model in any location in the world, obtaining a more comprehensive analysis only calibrating the model with the initial monitoring. Moreover, simulation can help to match shelter types to appropriate climates and effective use and it may be a strategic tool for aid decisions.

### Evidence supporting future studies

There are several limitations in the assessment of shelter impact on health. Collected data on indoor environment of shelters and self-perceived health of shelter occupants often failed to convey the impact of shelter on health (Nix et al. 2020) due to lack of a transdisciplinary approach. Accurate, representative, relevant, generalisable, attributed, methodological clarity are the six factors identified by ALNAP (Christoplos et al. 2017) that constitute reasonable evidence. However, obtaining good evidence in the humanitarian context is challenging, due to the instability of the context and the difficulties to obtain medical data mainly due to ethical issues. Good evidence is also supported by an appropriate methodology.

Most of the studies are based on interviews and self-reported morbidity linked to shelter problems and self-reported shelter issues. Self-reports have been used in several studies (Al-Khatib et al. 2005; Al-Khatib and Tabakhna 2006), but they do not include accurate indicators to measure health impacts of shelter project and proper shelter measures. This can lead to a misunderstanding of the health outcomes. For example, Tustingi (2020), using national survey data on shelter and child health, found a positive association between shelter improvements and reduction in diarrhoea, anaemia, and malaria. However, when the author asked for the impact of improved shelter on acute respiratory infections (ARI), a statistically significant association could not be found. This may be due to the fact that the study did not analyse any measure of 'shelter improvements' such as windows or ventilation, which were only measured by self-report householders. An example of good practice research assessing the impact of shelter interventions on health was proposed by Nix et al. (2020). The author, in a study on the impact of shelter on health in an informal settlement in Delhi, utilised a mixed-method approach which drew on different disciplines from the built environment, health, social science and included households and communities, followed by a risk assessment framework identification. This methodology seems significant for capturing evidence on shelter conditions through an evaluation of shelter-related risk from a building perspective.

‬‬‬‬‬‬‬‬‬‬‬‬‬‬‬‬‬‬‬‬‬‬‬‬‬‬‬‬‬‬‬‬‬‬‬‬‬‬‬‬‬‬‬‬‬‬‬‬‬‬‬‬‬‬‬‬‬‬‬‬‬‬‬‬‬‬‬‬‬‬‬‬‬‬‬‬‬‬‬‬‬‬‬‬‬‬‬‬‬‬‬‬‬‬‬‬‬‬‬‬‬‬‬‬‬‬‬‬‬‬‬‬‬‬‬‬‬‬Nonetheless, the residents’ perspective results are fundamental to understand the local perspective and the needs in particular concerning thermal comfort and privacy. Data gathering through interviews, focus group discussion and field observation are fundamental to assess the habits of people and to understand how to achieve a healthier indoor environment in displacement. Further evidence is vital to understand broader impacts and to assess current shelter conditions across developing countries and a transdisciplinary approach is necessary to provide a holistic understanding of the impact of shelters on health and to develop an effective methodology of assessment.

### Limitations in the assessment of the impact of shelters on health

Several methods can be used to measure health status such as clinical assessments, large scale demographic health surveys (DHS) and diagnostic investigations (Prinja et al. 2012). Self-reports are one of the most widely used methods in research, nevertheless, they are a cause of bias especially in developing countries. Some doubts arise in particular for cross-population comparison, because respondents can be influenced by social experience, education and culture which are specifical for each country or region. As a consequence, the validity and objectivity of self-reported health status may be misleading and has been criticised from an anthropological perspective (Amartya Sen 2002).

Overcoming the problem of positional objectivity may involve the use of 'decomposition analysis', a regression-based method which helps to determinate inequalities in the given answers and decompose these inequalities into determinants. A calibration of respondents’ self-report can also be done through hypothetical scenarios, known as vignettes. Vignettes make possible to compare self-reports from people with different economic, social and cultural characteristics.

For health assessment, the use of self-report should be combined with the observations of doctors, but gathering medical data from displaced populations is a significant challenge due to the unpredictability of the scenario and difficulties in diagnosing in displaced populations (World Health Organization 2018c). Al-Khatib (Al-Khatib et al. 2003, 2005; Al-Khatib and Tabakhna 2006) used self-reports to assess both health status and shelter quality. Participants, for example, have observed and assessed the presence of mould or visible fungal growth. More commonly, self-reports are used to understand occupants’ perception of thermal comfort (Albadra et al. 2017; Thapa et al. 2018, 2019) with questions concerning thermal sensation and thermal preference. However, as in the case of self-reported health, self-reports on shelter quality may be influenced by cultural factors or inadequate building knowledge. To overcome the problems associated with the bias of self-reporting, field observations and shelter measures can help to assess shelter quality. The participation of households remains essential and part of the process of improving design (Hamilton 2012; Dabaieh and Alwall 2018). In addition, this method has the advantage to report the longer perspective of the households. Therefore, a more objective and standardised approach might include the combination of interviews with shelter monitoring and measurements and shelter modelling.

## Conclusion

Considering the growing number of refugees and IDPs and the need to provide them with a healthy shelter, this review has compared different studies to describe the main themes of the existing literature and to inform future research.

Although the humanitarian sector has released a significant number of guidelines, they rarely provide clear guidance concerning shelter and health. Covered living area per person is the most highlighted standard in each handbook and manual. However, this indicator does not represent a complete measure of the shelter adequacy and other indicators such as thermal comfort or materials are overlooked. There is also a lack of uniformity in the terminology adopted by the humanitarian actors and various authors, especially when describing shelters or housing, which often leads to misinterpretation. It is recommended that future guidelines should provide clear guidance on healthy shelter. Additionally, a more uniform vocabulary is needed in order to facilitate the comparative analysis between different case studies, which is currently challenging.

The systematic bibliometric analysis presented in this study was designed to shed light on a field of research concerning the impact of shelter on human health, which is deeply overlooked and not regulated by a precise terminology and guidelines. However, this investigation demonstrates that there is a limited number of studies concerning shelter and health and even a more limited number of studies directly designed to investigate the relationship between shelter and health. Field studies mainly relied on self-reporting about shelter conditions and health outcomes, without measuring shelter quality in terms of indoor conditions. On the other hand, there is still a knowledge gap regarding the measuring system to adopt when analysing shelter quality. For example, in the evaluation of thermal comfort three main approached can be adopted, but rarely did authors use the combination of these approaches, which seem to provide valid data.

Several studies have demonstrated the correlation between overcrowding and infectious disease and respiratory diseases. Other researchers observed that thermal comfort and air quality inside shelters have a significant impact on refugees’ and IDPs’ health. They also observed that through the implementation of smaller retrofits, it is possible to provide displaced populations with a healthier indoor environment. None of the researchers have conducted a holistic analysis of the indoor environment of shelters, but instead analysed different factors, such as air quality or thermal comfort separately. From their results, it is still unclear how shelters determine the development of certain diseases. For instance, there is evidence that poor air quality increases the incidence of respiratory disease and that overcrowding is linked to respiratory infections and diarrheal disease. However, none of the studies provides a measure of this relationship. Also, poor thermal comfort was found to be a threat for refugees and IDPs, but very little research provides evidence of it. Therefore, further research is needed first to provide evidence of the impact of shelter IAQ and thermal conditions on human health and then to establish a clear methodology for investigating the effects of shelter on health.

Self-reports on shelter conditions and health outcomes have been primary adopted as the main methodology. Especially in the assessment of thermal comfort, personal questionnaires played an important role to define occupants’ comfort and desires. On the other hand, investigation through modelling and field measurement have been demonstrated to be reliable approaches. A more holistic approach in investigating shelters IAQ and thermal comfort should be adopted to understand the impact on human health. Furthermore, the heterogeneity of case studies presented in this review demonstrates that further research is needed to provide new case studies with more similarities to be compared.

## Data Availability

Not applicable.

## Abbreviations

ARI: Acute respiratory infections
DHS: Demographic Health Survey
IAQ: Indoor air quality
ICRC: International Committee of the Red Cross
IDP: Internally displaced person
IEQ: Indoor environmental quality
IFRC: International Federation of Red Cross and Red Crescent Societies
PMV: Predicted mean vote
TSV: Thermal Sensation Vote
UNHCR: United Nations High Commissioner for Refugees
URTI: Upper respiratory tract infection
VOCs: Volatile organic compounds
WaSH: Water and sanitation hygiene
WHO: World Health Organization
